# Influence of Radiotherapy on Ossification of Vascularized Osseous Reconstruction of the Jaw: A Radiological Retrospective Cohort Study Based on Panoramic Radiographs

**DOI:** 10.3390/jcm11175041

**Published:** 2022-08-27

**Authors:** Maximilian Gottsauner, Clara Fehrer, Steffen Spoerl, Johannes Schuderer, Florian Zeman, Mathias Fiedler, Michael Maurer, Torsten E. Reichert, Tobias Ettl

**Affiliations:** 1 Department of Oral and Maxillofacial Surgery, University Hospital Regensburg, 93053 Regensburg, Germany; 2 Center for Clinical Studies, University Hospital Regensburg, 93053 Regensburg, Germany

**Keywords:** ossification, radiotherapy, microvascular reconstruction, jaw, mandible, fibula

## Abstract

Background: The aim of this study was to evaluate the impact of irradiation and time of irradiation on the ossification of jaws reconstructed with free bone grafts. Methods: In total, 100 reconstructions of the jaw were retrospectively evaluated for ossification between bone segments by two raters based on postoperative panoramic radiographs (immediate postOP, approximately 6, 12 and 24 months follow-up). Three subgroups were divided according to the time of irradiation: preoperative radiation therapy (n = 41), postoperative radiation therapy (n = 26) and patients without any radiation therapy (n = 33) as the control group. Ossification time and influencing factors were documented. Results: The fastest ossification with a median of 304 ± 37 days was observed (*p* < 0.001) in the nonirradiated control group. No significant difference (*p* = 0.087) in ossification was found between the pre- (447 ± 136 days) and postoperative (510 ± 112 days) radiation groups. Ossification between two graft segments (336 ± 38 days) showed significantly (*p* < 0.001) faster ossification than between the original and grafted bone (448 ± 85 days). Moreover, closer initial contact between the segments resulted in faster ossification (*p* < 0.001). When analyzing cofactors, tobacco consumption was the only negative factor aggravating ossification (*p* = 0.006). Conclusion: Head and neck radiation corresponded with the impaired and prolonged ossification of jaw reconstructions with free bone grafts. There was no difference in ossification if radiotherapy was performed before or after reconstructive surgery. A close bony contact was particularly important for ossification between the original and grafted bone.

## 1. Introduction

Oral squamous cell carcinomas invading adjacent jaw bones often require jaw resection and postoperative radiotherapy. There is an ongoing discussion for the ideal moment of jaw reconstruction. Some authors recommend immediate reconstruction due to simplified vessel preparation and anastomosis. Others advocate secondary jaw reconstruction after a reasonable tumor follow-up. Secondary reconstruction, however, has disadvantages caused by side effects of radiotherapy, and can impede bony reconstruction [1,2]. This also concerns bony reconstructions due to osteonecrosis after primary radiotherapy [3,4].

Noncancer-associated bony microvascular reconstructions of the jaw include defects after severe maxillofacial traumata, advanced inflammatory osteomyelitis or after the resection of benign tumors, such as ameloblastoma [5,6]. The early surgical treatment of MRONJ can prevent its severe progress, including the resection and reconstruction of the jaw [7].

Currently, especially to span greater distances, the microvascular osteocutaneous free fibula flap has been established as the first option for most reconstructions of the mandible. Multiple segmentations of bone grafts allow for a proper restoration of the mandibular curvature. The reliable skin paddle enables this free flap to safely cover soft tissue defects too [8]. Rarely, due to arteriosclerotic strictures and anatomic variants of the lower leg vessels, the versatile fibular flap cannot be raised [9]. In that case, the free iliac crest bone flap and the scapular flap serve as alternative options of bony jaw reconstruction. For all osseous reconstructions, the final aim is the stable ossification of the residual jaw followed up with the complete removal of all metal material [10]. The complete removal of osteosynthetic material can prevent secondary complications related to the plates [11]. Computer-assisted planning and usage of patient-specific implants are supposed to increase the close adaption of the segments, enabling enhanced and complete bone healing compared to a freehand approach [12]. The insertion of dental implants, eventually combined with additional augmentation techniques, should lead to a full prosthetic rehabilitation of the patient [13]. The implantation of CAD/CAM-planned titanium meshes on the primary reconstructed mandible can help to gain height and shape of the transplanted fibula [14].

Significant side effects of radiation are seen during bone healing, particularly in susceptible parts such as the nonmineralized bone matrix. Among others, functioning osteocytes and osteoblasts are markedly reduced [15]. Moreover, the vitality of irradiated vascular tissue and periosteum is negatively affected. Processes such as long-term fibrotic tissue changes can be observed, resulting in severe alterations of tissue and functional limitations for the patient [16,17]. Clinically, these functional changes imply, for example, reduced success rates of implant-based prosthetic restorations after radiotherapy [18,19], with higher amounts of crestal peri-implant bone loss in irradiated jaw areas [20]. Several investigators have shown the feasibility of mandibular reconstruction after irradiation with the use of free fibular grafts, although rates of infections and wound complications increased [21]. In contrast, little data exist about the effect of irradiation on bone graft ossification, particularly with regard to the specific impact of pre- or postoperative radiotherapy.

The aim of this retrospective study was to evaluate the ossification of the jaw, reconstructed with free vascularized bone grafts, whilst comparing between nonirradiated patients and patients who underwent pre- or postoperative radiotherapy.

## 2. Patients and Methods

The study design was approved by the ethics committee of the University of Regensburg (ref. 18-1131-104) in accordance with the Helsinki declaration and its later amendments or comparable ethical standards.

### 2.1. Patients

Between 2008 and 2018, 104 operations for free microvascular bony reconstructions of the jaw were performed in the Department of Oral and Maxillofacial Surgery at the University Hospital in Regensburg. Four cases were excluded due to a total loss of the transplant, so the ossification could not be evaluated. Therefore, 100 cases were included in the study. Three different types of microvascular bony reconstruction were performed. The predominant type of reconstruction was the fibula flap (n = 86), followed by the free iliac crest (n = 11) and scapular (n = 3) flaps. Three patients received two different microvascular bony transplants, and each case was examined separately. Technically, osteotomies were performed with the use of an oscillating saw. For osteosynthesis, load-bearing reconstruction plates were used in all cases. In CAD/CAM cases, patient-specific implants provided by the manufacturers were inserted.

The following data were collected: age, gender, diagnosis, radiation history, location of malignancy, preoperative CAD/CAM planning, defect classification after Jewer and Boyd [22], type of bony reconstruction, amount and length of the bone segments, duration in ICU, duration of entire hospitalization, smoking status and alcohol abuse history.

The cohort was divided into three subgroups (Table 1): (1) postoperative radiotherapy (postORT) with jaw reconstruction performed before radiotherapy (n = 26); (2) preoperative radiotherapy (preORT) with jaw reconstruction performed after radiotherapy (n = 41); and (3) patients without any radiation therapy (n = 33) as a control group (noRT).

**Figure 1:**
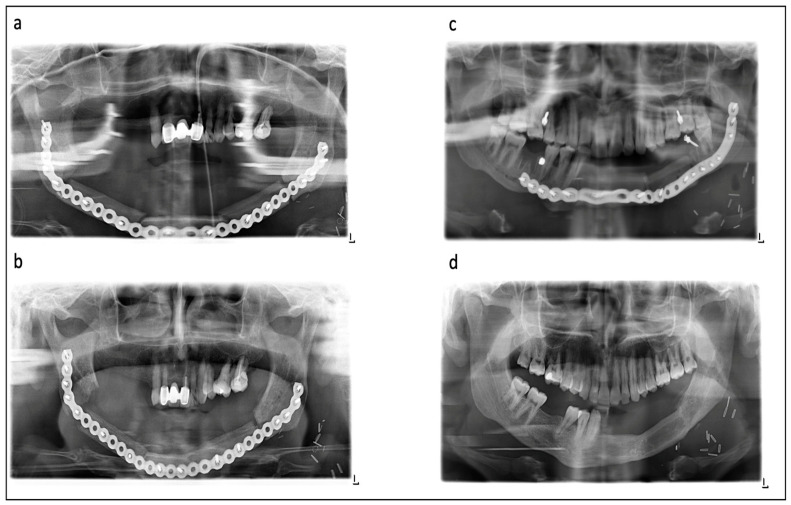
Various examples for ossification in different radiographs: (**a**,**b**) show patient after preoperative radiotherapy, consecutive osteoradionecrosis of the mandible and reconstruction with a 3-segmented fibula. (**a**) Initial postsurgical radiograph with good contact between fibular segments in left canine area, moderate contact in right canine and right angle area and no contact in left angle area. (**b**) After 6 months, complete intersegmental ossification and nonossification between segments and original bone. (**c**,**d**) show young patient after gunshot wound and reconstruction with a two-segmental fibula. (**c**) Initial postsurgical radiograph with good contact between fibula segments and graft/native bone. (**d**) After 1 year, complete ossification of all contacts allowed for removal of the reconstruction plate.

Panoramic radiographs at approximately the 10th postoperative day and the 6th, 12th and 24th postoperative months were examined. After full ossification at each contact point, no further examinations were conducted. Two raters (C.F. and M.G.) with long-term clinical experience in dentistry and maxillofacial surgery independently examined all radiographs. The quality of intrasurgical contact between the bone graft and original bone, as well as between graft segments, was evaluated on initial postoperative radiographs (the 10th postoperative day). The quality of contact was divided into "no contact" (radiographic gap > 2 mm), "moderate contact" (gap ≤ 2 mm) and "good contact" (no gap) (Figure 1). The sites of segmentations (the symphysis, canine, corpus, angle and maxilla), segment number and segment length of the bone graft were evaluated too. All the results were correlated to the healing time after surgery.

Follow-up radiographs during healing were evaluated for quality of ossification as follows (Figure 1): no ossification (no sign of ossification vertically between the segments), partial ossification (less than 50% ossification between the segments) and complete ossification (more than 50% ossification between the segments). In the case of a diverse evaluation between the raters, a review was performed by two additional senior experts and a final statement was expressed.

### 2.2. Data Analysis

Continuous data were presented as mean ± SD or as median (first quartile and third quartile) depending on the underlying distribution. Data were compared between groups by using an analysis of variance (ANOVA) or the nonparametric Kruskal–Wallis test. Time to ossification was analyzed by using Kaplan–Meier plots and log-rank tests, as well as a multivariable Cox proportional hazards model, including all significant variables of the univariable analyses. Hazard ratios (HRs) and corresponding 95% confidence intervals (95% CIs) were reported as effect estimates. A *p*-value < 0.05 was considered statistically significant for all tests. All statistical analyses were performed using IBM SPSS Statistics, version 26 (IBM Corp., Armonk, NY, USA), while Kaplan–Meier plots were generated by using R, version 4.1.2 (The R Foundation for Statistical Computing, Vienna Austria).

## 3. Results

The first panoramic radiograph was performed at approximately the 11th postoperative day (11 ± 8) as a starting point, and was used for the evaluation of the primary contacts between segments. Mean surveillance periods were at three different postoperative check-up dates. The first was at approximately the 7th month (205 ± 43 days), the second at approximately the 13th month (405 ± 102 days) and the last control X-ray was performed at approximately the 24th month (733 ± 112 days) after surgery. Overall, 807 points of contact between the segments were documented in all panoramic radiographs. In detail, 292 (initial postOP), 209 (6 months), 201 (12 months) and 105 (24 months) contact points were evaluated. The rates matched in 640 cases, which led to an inter-rater accuracy of 79%. In 96% (n = 26) of the cases in the postORT subgroup, the reconstruction was combined with a neck dissection, compared to 61% (n = 25) in the preORT subgroup and 33% (n = 11) in the noRT group. Therefore, the duration of the operation was within 625 ± 164 min, significantly longer in the postORT subgroup than in the preORT subgroup *p* < 0.001 (491 ± 155 min) and the noRT group *p* = 0.002 (501 ± 126 min). The average postoperative stay in the ICU showed no significant difference; the postORT subgroup stayed 4.4 ± 2.9 days, the preORT subgroup stayed 4.4 ± 3.6 days and the noRT group stayed 3.7 ± 3.0 days. Regarding the overall hospitalization time, both radiation therapy groups showed significant difference to the control group (12.9 ± 5.0 days); postORT subgroup (21.2 ± 8.9 days) with *p* < 0.001 and preORT subgroup (17.0 ± 8.3 days) with *p* = 0.012. The parameters of defect size showed no significant difference between the groups. The postORT subgroup had the largest average defects, 103.7 ± 29.5 mm, followed by the noRT group with 97.7 ± 36.6 mm and the preORT subgroup with 95.7 ± 37.1 mm.

An analysis of CAD/CAM-planned cases versus freehand-performed reconstructions of the jaw showed no significant difference in ossification *p* = 0.882.

### 3.1. Overall Ossification between Pre- and Postoperative Radiation Therapy Compared to the Control Group

**Figure 2:**
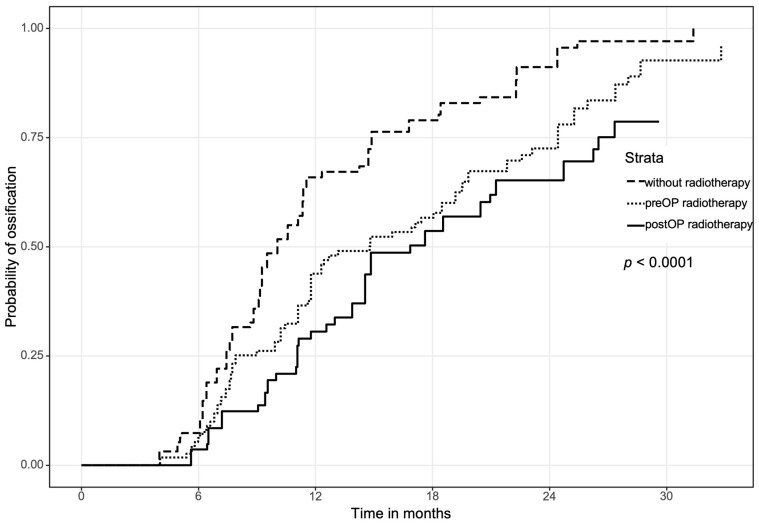
Overall complete ossification for every point of contact depending on radiotherapy.

The overall median for complete ossification at the point of contact was 510 (IQR 398–622) days for the postORT subgroup, followed by the preORT subgroup with 447 (IQR 311-583) days. The fastest ossification was observed in patients without any radiation (304 IQR 267–341 days). Patients of both groups who underwent radiation showed significantly slower complete ossification *p* < 0.001 than nonirradiated ones (Figure 2). Between the two subgroups with radiation therapy, no significant difference (*p* = 0.087) in overall ossification was observed.

### 3.2. Ossification Concerning the Bone Quality

**Figure 3:**
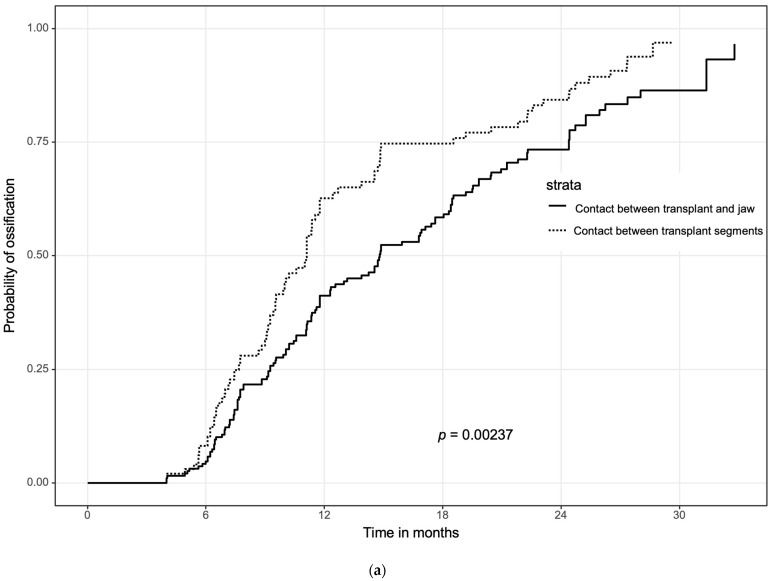

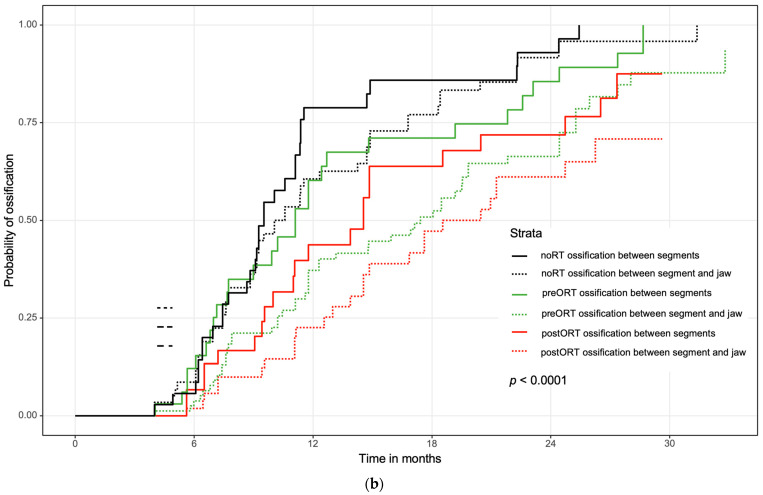
(**a**) Ossification concerning the contact between two segments of the transplant and between one segment of the transplant and the original bone of the jaw. (**b**) Ossification concerning the contact between two segments of the transplant and between one segment of the transplant and the original bone, subdivided regarding time and event of radiation therapy. Bone healing was faster between graft segments compared to segment/jaw contacts in both preORT and postORT groups.

The median ossification of contact points between two segments of the microvascular transplant showed within 336 (IQR 298–374) days and with *p* = 0.002, a significantly faster ossification (*p* = 0.002) than the ossification between one segment of the transplant and the original recipient bone within 448 (IQR 363–533) days (Figure 3a). 

These results were subdivided regarding nonirradiated and pre- and postoperative radiotherapy groups (Figure 3b).

After testing all subgroups against each other (Table 2), the control group with nonirradiated patients achieved the fastest ossification without any significant difference between the two segments of the transplant and one segment of the transplant and the original bone (*p* = 0.334). In patients with preoperative radiotherapy, the contact area between two transplant segments showed a similar ossification rate compared to the nonirradiated control group (*p* = 0.659 and *p* = 0.128), whereas ossification between the graft and native jaw bone was significantly worse (*p* < 0.001). In patients with postoperative radiotherapy, ossification between the graft segments was significantly slower than in the control (*p* = 0.040 and *p* = 0.003), and was slower than the preORT group, but ossification was still faster than ossification between the graft and native jaw bone without reaching significance (*p* = 0.094). Overall, ossification was worst between the graft and jaw bone in both the preORT and postORT groups (Figure 3b).

### 3.3. Regional Ossification

**Figure 4:**
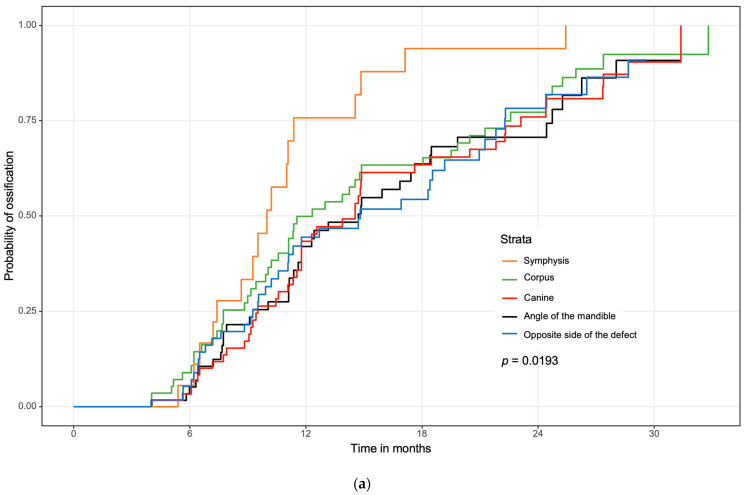

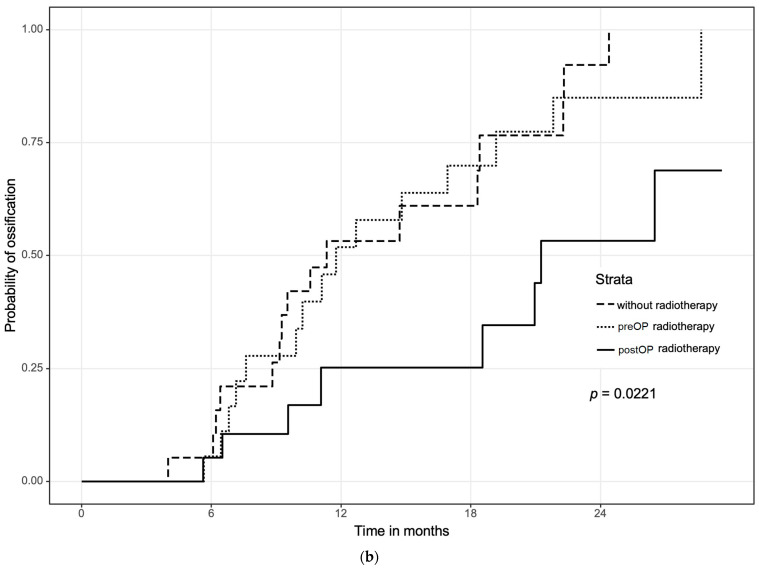
(**a**) Overall locations of the mandible. Reconstructions of the maxilla were excluded in this figure due to a lack of data. (**b**) Separate evaluation of the contralateral (opposite) side. Significantly worse ossification in the postORT group.

Regarding different jaw regions, the symphysis showed fastest overall complete ossification with a median of 302 (IQR 264–340) days (Figure 4a). This value was significantly faster than all other examined regions (*p* = 0.039 to *p* = 0.003).

Regarding contact localizations and noRT, pre- or postoperative radiation therapy, only the region of the corpus and the opposite side (Figure 4b) showed significant results between the subgroups. In the region of the corpus, the nonirradiated patients showed significantly faster complete ossification compared to patients with postoperative (*p* < 0.001) and preoperative (*p* = 0.009) radiation therapy. Between the pre- and postoperative subgroups, no significant difference could be observed. The opposite side was defined as the contact point contralateral to the main defect and also contralateral to the target volume in case of irradiation. In comparison to the postORT subgroup, the noRT (*p* = 0.007) and the preORT (*p* = 0.036) subgroups showed significantly faster complete ossification at the contralateral side.

### 3.4. Influence of Initial Contact between the Bone Segments on Ossification and Analysis of Multiple Variables Regarding Ossification

A good initial contact between the different bone segments led to significantly faster complete ossification than a modest contact (*p* < 0.001). The median for complete ossification at no initial contact was 448 (IQR 349–748) days, 346 (IQR 275–520) days for moderate initial contact and 334 (IQR 225–533) days for good initial contact.

Moreover, age and gender showed no significant influence on ossification, while smoking showed a significant adverse influence on the ossification of microvascular reconstruction in the head and neck region (HR (95% CI), 1.48 (1.12; 1.95), *p* = 0.006).

In a multivariable model, only postoperative radiation therapy showed significantly slower ossification with *p* = 0.002, and contact between segments of the bone graft with *p* = 0.035 (Table 3).

## 4. Discussion

There is an ongoing discussion whether microvascular reconstruction should be performed immediately after tumor resection or be delayed, for example, after a one-year follow-up in case of malignancy [1,2,3,4]. Besides other issues, such as tumor control and an impeded secondary surgery due to scarring and abnormal vessel anatomy after prior surgery and/or radiotherapy, one further issue is the question of ossification and the proper healing of transplanted bone grafts. Therefore, the aim of this study was to analyze the influence of radiotherapy on the ossification of microvascular bony reconstructions. A cohort of 100 patients was screened retrospectively and postoperative panoramic radiographs evaluated regarding the process of ossification.

As expected, the ossification of the free bone graft at the examined contact areas was fastest in patients who did not receive any radiotherapy at all (noRT, control group), followed by patients with preoperative radiotherapy (preORT), and patients with postoperative radiotherapy (postORT). According to the results of this study, full ossification in nonirradiated patients could be expected after 10 months, while complete ossification in patients with prereconstruction radiation took 15 months and, in patients with postreconstruction radiation, ossification took approximately 17 months.

The effect of therapeutic radiation on tissue has been known for decades [23]. According to animal models, radiation has a major impact on composition, cell matrix and remodeling processes. Radiation therapy can lead to enhanced activity among osteoclasts with a loss of trabecular bone, slower bone turnover and the accumulation of a less stable matrix [15]. This effect was observed in periosteal and endo-osteal parts with decreasing mineralization and increasing collagen crosslinks [17]. In canine models, nonvascularized and vascularized rib grafts were examined regarding union after radiation. Ossification was seen in both groups without radiation, whereas only the vascularized graft achieved ossification in the irradiated group [24]. A similar dog model showed significant lower stability against bending and torsion forces in vascularized bone grafts after radiation [25]. Interestingly, the loss of trabecular bone was not only detectable in the irradiated area, but the contralateral side was also affected, and intermediate changes in bone composition were measured [26].

Jegoux et al. prepared a survey regarding the effects of radiation on bone healing and the reconstruction of oral and maxillofacial surgery. Besides the already mentioned changes in the molecular structure of the bone, the cellular remodeling of irradiated bone was described. This could lead to the minor vascularization of the trabecular bone and the periosteum. Moreover, radiation-induced fibrosis and metabolic shift in the irradiated tissue can worsen the imbalance. Finally, severe irreversible damage of the bone structure can result in the osteoradionecrosis (ORN) of the jaw [16]. Tumor location, radiation dose and volume of the irradiated jaw are known risk factors that could lead to ORN [27,28]. Besides higher postoperative complication rates after microvascular free flap reconstructions, the overall survival of the flaps did not suffer significantly [29]. Most common complications were fistulas, hardware plate exposure and flap wound infections. The reconstruction of the mandible due to ORN with a free flap fibula was postulated as the gold standard [30]. The effect of radiation on the ossification of the bony reconstruction was not part of the cited surveys. Missing or delayed ossification was clearly estimated as a minor complication. Nevertheless, regarding plate exposure or infections, the immediate removal of osteosynthesis may become inevitable. The majority of published papers debating the effect of radiation on vascularized free flaps have tried to clarify the complication and failure rates.

The recipient vessel in the irradiated head and neck region did not trigger the loss of vascularized flaps [31]. Besides more challenging surgical conditions, the complication rates showed no statistical difference [32,33,34]. Even the timing between pre- and postoperative radiotherapy had no impact on flap survival [35]. Otherwise, some surveys, for free flaps in general, observed a slightly higher failure rate [36,37]. Even the risk of osteosynthesis plate-related complications and radiotherapy has been discussed controversially in the literature [11]. Due to the high success of free flap surgery, these contradictions could be explained by various factors, such as individual planning and dosage of radiotherapy [38]. Patients with a complete loss of transplant were excluded in the cohort. This major complication would negate the research of the ossification of free flaps.

Gordin et al. examined the impact of multiple courses of radiotherapy on microvascular free flap reconstructions. Although the risk of ORN and healing complications regarding the irradiated wound bed rose, the surgical technique was feasible [39].

After analyzing pre- and postradiotherapy digital panoramic radiographs, a loss in trabecular microstructure and mandibular bone mass was documented [40]. The analysis was only conducted unilaterally, so no prediction about the opposite site was possible.

The results of the current study also underlined the clinical and radiological impact of irradiation on bone healing, but, of course, no statement on histological transformation could be determined due to the limitations of the radiographic analysis. In this study, we investigated the impact of radiotherapy performed before jaw reconstruction (preORT) to radiotherapy that followed after jaw reconstruction (postORT). As mentioned above, ossification was slower in the postORT group compared to the preORT group. For the interpretation of these results, one must keep in mind that all contact points (807 overall) between grafted bone and native jaw were evaluated. A closer consideration of the contact areas in this preORT group revealed significantly better ossification between graft segments than between transplanted and irradiated native bones. In this subgroup, ossification between grafted bone segments was comparable to nonirradiated patients. This result was not a surprise, as bone healing between the graft segments was not impaired by prior radiotherapy at all. Meanwhile, the contact between irradiated native and nonirradiated grafted bone showed slower ossification. However, ossification between graft segments was also faster in the postoperative radiation therapy subgroup compared to graft/jaw contacts without reaching significance. One reason may also be a better matching contact area between graft bones compared to the contact area between graft bone and, for example, the thinner bone of the mandibular angle. The current study also showed that the closer the initial contact between bone segments, the faster the ossification could take place in all subgroups. Nevertheless, contact between two graft segments showed significantly faster complete ossification than the contact points between bone graft and original bone. Swendseid et al. showed a similar effect, including less dependency on distance between graft segments for complete ossification [41]. In this context, the data from our multivariate analysis emphasized that radiotherapy as well as the type of bone contact (graft/native vs. graft/graft) showed significantly stronger impacts on ossification and the initial quality of bone contact. This meant that even segments with a gap of 2 mm or more could sufficiently ossify as long as there was no radiotherapy.

A comparison of intergraft ossification between preORT and postORT showed slower healing in the postORT group, but, surprisingly, without reaching statistical significance. The bone healing of vascularized grafts may not have depended on radiotherapy in the same way as the healing between the bone graft and original bone of the jaw.

Regarding the location of the contact areas, the angle of the mandible presented a slower ossification compared to the contacts in the corpus and, particularly, to the symphysis area. In areas of slower ossification, overlapping a split fibula with the mandibular angle may improve bone healing and stabilize the gap.

On the contralateral area to the main defect, only the postORT group showed significantly slower ossification. This effect may have been provoked by an enlarged field of radiation near the contact point between the graft and original bone due to closer initial tumor resection borders.

The study had several limitations. First of all, its retrospective nature posed the risk of incomplete data and bias in data documentation and the statistical analysis. A major drawback regarding radiological evaluation was the lack of a 3D evaluation of ossification. The use of a multislice CT scan or a cone-beam CT may have provided more precise data. In clinical routine, however, plain panoramic radiographs were sufficient for most questions of ossification or hardware failure. At our institution, 3D scans were reserved for tumor follow-up or specific bone diagnostics during recent years. Finally, the used reconstruction plates occasionally covered the gaps, sometimes hindering evaluation.

## 5. Conclusions

This study demonstrated that radiotherapy led to delayed ossification regardless of a pre- or postoperative regime. In particular, the union between the original bone and transplant was critical, and a close bony contact should be achieved here. Ossification between graft segments was favorable, even in patients with previous irradiation. The indication for the microvascular reconstruction of the jaw was not determined by pre- or postradiotherapy, but by other factors such as defect size and primary diagnosis.

## Figures and Tables

**Table 1:** Cohort characteristics. (**a**) General information of all three groups. (**b**) Primary diagnosis and location for all three groups. (**c**) Defect classification and types of microvascular flap utilized.

	**Postoperative Radiotherapy (n = 26)**	**Preoperative Radiotherapy (n = 41)**	**Nonirradiated (Control Group) (n = 33)**
(**a**)			
Male/female, n (%)	20 (77%)/6 (23%)	26 (63%)/15 (37%)	9 (58%)/14 (42%)
Age in years, median (IQR)	59 (51, 64)	59 (51, 67)	53 (44, 63)
Radiation dose in Gy, median (IQR)	64.9 (60.0, 66.6)	66.0 (60.0, 70.0)	none
Tobacco, n (%)	20 (77%)	27 (66%)	15 (46%)
CAD/CAM planning, n (%)	0 (0%)	7 (17%)	4 (12%)
Neck dissection, n (%)	25 (96%)	25 (61%)	11 (33%)
(**b**)			
**Primary Diagnosis**			
Malignant Tumor	26	18	16
Osteoradionecrosis	0	23	0
MRONJ	0	0	2
Unspecific Osteomyelitis	0	0	10
Gun shot wounds	0	0	3
Benigne Tumor	0	0	2
**Side of malignancy (n = 62)**			
Lateral Floor of Mouth/Mandibel	25	16	14
Maxilla	0	1	5
Skin	0	1	0
**Side of primary malignancy** **in ORN (n = 23)**			
Lateral Floor of Mouth/Mandibel		5	
Anterior Floor of Mouth/Lower Lip		1	
Tongue		2	
Pharynx/Tonsille/Root of Tongue		9	
Larynx		1	
Maxilla/Nose/Sinus maxillaris		2	
Parotid Gland		1	
More than one Location		2	
(**c**)			
**Defect Classification of Boyd and Jewer**			
C	1	0	1
H	0	1	1
HC	0	0	2
HCL	0	0	1
HL	0	1	0
L	7	19	6
LC	5	7	9
LCL	13	12	8
Maxilla	0	1	5
**Typ of flaps**			
Fibula	24	36	26
Iliac crest	1	5	5
Scapula	1	0	2

IQR, interquartile range; CAD/CAM, computer-aided design/computer-aided manufacturing; MRONJ, medication-related osteonecrosis of the jaw; ORN, osteoradionecrosis.

**Table 2:** *p*-values of ossification between bone graft and original bone of the jaw regarding subgroups noRT, preORT, and postORT (significant *p*-values are marked **bold**).

	postORT and Contact between Graft and Original Bone n = 53	preORT and Contact between Graft and Original Bone n = 82	noRT and Contact between Graft and Original Bone n = 62	postORT and Contact between Graft Segments n = 30	preORT and Contact between Graft Segments n = 33	noRT and Contact between Graft Segments n = 35
**postORT** and contact between **graft and original bone**		0.164	**<0.0001**	0.094	**0.001**	**<0.0001**
**preORT** and contact between **graft and original bone**	0.164		**<0.0001**	0.601	**0.015**	**<0.0001**
**noRT** and contact between **graft and original bone**	**<0.0001**	**<0.0001**		**0.040**	0.659	0.334
**postORT** and contact between **graft segments**	0.094	0.601	**0.040**		0.135	**0.003**
**preORT** and contact between **graft segments**	**0.001**	**0.015**	0.659	0.135		0.128
**noRT** and contact between **graft segments**	**<0.0001**	**<0.0001**	0.334	**0.003**	0.128	

**Table 3:** Multivariable Cox regression model on ossification.

	HR	95%-CI		*p*-Value
**Radiotherapy**				
Nonirradiated	Reference			
Postoperative radiotherapy	0.529	0.353	0.792	0.002
Preoperative radiotherapy	0.728	0.514	1.031	0.074
**No tabacco abuse**	1.350	0.980	1.860	0.066
**Contact between transplant segments**	1.391	1.024	1.890	0.035
**Amount of segments of the transplant**				
1 segment	Reference			
2 segments	1.685	1.113	2.552	0.014
3 segments	1.290	0.820	2.031	0.271
**Quality of initial contact**				
No initial contact	Reference			
Moderate initial contact	1.055	0.473	2.351	0.896
Good initial contact	1.523	0.705	3.292	0.285
